# Laparoscopic Umbilical Hernia repair in male patients with abdominal obesity

**DOI:** 10.12669/pjms.38.7.6470

**Published:** 2022

**Authors:** Abdulrahman Saleh Al-Mulhim, Abdul Qadeer Memon

**Affiliations:** 1 Dr. Abdulrahman Saleh Al-Mulhim, FRCSI, FICS, FACS. Professor of Surgery, Department of Surgery, King Faisal University College of Medicine, Al-Ahsa 31982, Kingdom of Saudi Arabia; 2 Dr. Abdul Qadeer Memon, FCPS, FICS. Assistant Professor of Surgery, Department of Surgery, King Faisal University College of Medicine, Al-Ahsa 31982, Kingdom of Saudi Arabia

**Keywords:** Umbilical hernia, Obesity, Laparoscopic repair

## Abstract

**Objective:**

Obesity is a global health problem, and obese patients are subject to developing abdominal wall hernias. There are few prospective studies comparing the laparoscopic method of umbilical hernia mesh repair between abdominal obesity patients and normal abdominal waist patients. The aim of this study was to evaluate the short-term outcomes (operative time, early complications and hospital stay) in the patients having laparoscopic hernia repair with abdominal obesity.

**Methods:**

This prospective cohort study was conducted at King Fahad Hospital Hofuf, Kingdom of Saudi Arabia from June 2014 to June 2021. Fifty four (54) adult male patients with umbilical hernia were included in this study. The patients were divided into two groups: Group-A: Patients with abdominal obesity (n=26), and Group-B: Patients without abdominal obesity (n=28). All the patients underwent laparoscopic repair of umbilical hernia. The patients with abdominal obesity were defined as those having an abdominal girth more than 102 centimeters.

**Results:**

No significant differences were observed as related to age, co-morbidity and risk factors between the two groups. The statistically significant difference between the two groups observed was related to the mean operative time and the mean hospital stay.

**Conclusion:**

Laparoscopic umbilical hernia repair can be safely performed in abdominal obesity in male patients without an additional risk of complications.

## INTRODUCTION

The umbilicus is one of the potential weak areas of the abdomen. Umbilical hernia is a common disease and it represents 10% of all abdominal wall hernias.^1^ Obesity is an important health challenge in the world.^2^,^3^ and it is one of the major causes of increased intra-abdominal pressure which may result in developing an umbilical hernia.^4^

Obese patients are prone to developing abdominal wall hernias with/without all potential complications. A number of studies have found an association between operative difficulty in obese patients^5^ and post-operative complications.^6^ Recent studies have identified the impact of body mass index (BMI) on open and laparoscopic hernia surgery.^7^ Although, abdominal obesity is more important measure of central abdominal fat and is a better predictor of morbidity^8^, but no study has explored the effect of abdominal obesity on the outcome of hernia surgery. Abdominal obesity is the accumulation of visceral fat resulting in an increase in waist size and it is an indication of adverse metabolic outcomes independent of body mass index. The absolute waist circumference (>102 centimeters in men and >88 centimeters in women) are used as parameters of abdominal obesity.^9^

Our objective was to evaluate the short-term outcomes (operative time, early complications, and hospital stay) of laparoscopic hernia repair in patients with abdominal obesity.

## METHODS

This prospective cohort study was conducted at King Fahad Hospital Hofuf, Saudi Arabia from June 2014 to June 2021. The approval was got from the Research Ethics Committee of the King Faisal University College of Medicine (Ref No.: 2020-05-24, dated August 31, 2020). Fifty-four adult male patients with uncomplicated umbilical hernias were included in this study. The demographic data and outcome of the surgery of every patient were recorded in SPSS-22. The data included were age, clinical presentation, American Society of Anesthesiologists (ASA) score, co-morbidity, size of the defect of umbilical hernia, anesthetic duration, operative time, intraoperative complications, post-operative complications, post-operative pain, length of hospital stay, return to normal activity and recurrences. The results were expressed as mean, ± standard deviation and the statistical significance difference as *p*-value < 0.01. WHO classification was used for defining obesity (BMI=30kg/m^2^). The absolute waist circumference (>102 centimeters) was used for defining abdominal obesity.^12^,^13^ The waist circumference was measured at a level midway between the lowest rib and the iliac crest using the measuring tape.

Fifty four patients with umbilical hernias were divided into two groups depending upon waist circumference i.e. the abdominal obesity as a risk factor of possible complications: Group-A: Patients with abdominal obesity (n=26), and Group-B: Patients without abdominal obesity (n=28). All the patients underwent laparoscopic repair. The diagnosis of umbilical hernia was based on detailed clinical history, physical examination, and the necessary radiological investigations (ultrasound/computed scan). Base line and specific investigations for pre-anesthesia assessment were carried out. A preoperative abdominal CT-scan without contrast was routinely used in all patients to determine the abdominal obesity.

### Surgical procedure

The laparoscopic repair was performed under general anesthesia using a technique as originally reported for ventral hernias.^10^,^11^ The patients were placed on operation table in a supine position with both arms along the body, the surgeon at the left of the patient and the screen opposite to the surgeon. The pneumoperitoneum was made at 14 mmHg, established by veress needle introduced at Palmer’s point, which is a point 3cm below the left costal margin in the left mid-clavicular line. A 10mm, 30 optical cameras through 10mm trocar and other two 5mm trocars were placed as far away as possible from the hernia defect. The laparoscopic procedure was started by inspection of whole abdominal cavity. The adhesions surrounding the hernia defect, if found, were divided and the hernia contents were reduced. The mesh was measured with the abdomen deflated (the pneumoperitoneum at 8mmHg), allowing at least 5cm overlap beyond the borders of the fascial defect and applying knot with prolene suture at the four corners of the mesh. The mesh was hydrated by normal saline, rolled with the film inside and introduced into the abdominal cavity. A tiny stab skin incision was performed at four cardinal points, to pull each prolene knot of the mesh to stick it to the abdominal wall and fix it with absorbable tack by creating a double rounded ring. At the end, the abdomen was deflated under direct vision and the fascial defect of 10mm trocar was closed. A single dose of intravenous injection of broad-spectrum antibiotic was administered at the induction of anesthesia, followed by two postoperative doses. Deep vein thrombosis prophylaxis measures were taken in all the patients. These included the injection Clexane 40mg subcutaneously before operation and continued till the patients were discharged from the hospital, intermittent pneumatic compression device during the operation and encouraging early mobilization when the patients were fully awake.

The number of days of stay at the hospital was counted as the number of nights the patients were in the hospital postoperatively. Patients were allowed to take oral meals postoperatively after recovering from anesthesia. Patients were discharged when they were symptomatically better and advised to perform their routine daily activities. Post-operative pain and severity of pain was assessed daily during hospital stay using Visual Analogue pain Scale (VAS). The patients were followed-up at one, three, six and 12 months intervals after operation and evaluated for any complications and recurrences.

## RESULTS

Fifty four male adult patients with umbilical hernia underwent laparoscopic hernia repair. They were divided into two groups: Group-A: Patients with abdominal obesity (n=26), and Group-B: Patients without abdominal obesity (n=28) (Table-I). The overall mean age of the study sample was 39.4 ±3.2 years (range: 26-53). In Group-A, it was 38.9 ±8.8 years (range: 27-53 years) and in Group-B it was 39.5 ±4 years (range: 26-51 years). The mean waist circumference of Group-A patients was 117 cm, and that of Group-B was 79 cm. The mean BMI of Group-A was 36.3 and Group-B was 31.4. Group-A patients had more medical co-morbidities than Group-B. The diabetes mellitus and hypertension were present in 11.5%, and 3.8% in Group-A as compared to 7.1%, and 3.6% in Group-B respectively. There was no difference between the groups in terms of the American Society of Anesthesiologists (ASA) score. The mean symptomatic period of Group-A was 13 ± 1.1 months and the Group-B was 8 ±2.4 months. The defect size ranged between 2.3 cm and 9.6 cm and larger hernias were observed in Group-A as compared to Group-B.

**Table I:** Demographic data.

Characteristic	Group-A	Group-B	p-value
No. of Patients	26	28	> 0.01
Mean Age (rang) year	38.9 ±8.8 (27-53)	39.5 ±4 (26-51)	> 0.01
Mean waist circumference (cm)	117	79	< 0.01
Mean BMI (rang)	36.3 (31.4 – 39.7)	31.4 (26.8 – 32.9)	< 0.01
Co-morbidities			> 0.01
• Diabetes	11.5%	7.1%	
• Hypertension	3.8%)	3.6%	
Mean symptomatic period	13 ±1.1	8 ±2.4	< 0.01
Mean defect size (cm) (rang)	5.6 (4.5 – 9.6)	4.2 (2.3 – 4.7 )	< 0.01

All the patients were operated laparoscopically with a three-port approach. None were converted to open surgery and no intra-abdominal drains were placed. The mean operating time of Group-A was 75.4 minutes, while that of Group-B was 66.5 minutes. Hospital stay was two to five days (mean 3.3 days) in the Group-A and 2 to 4 days (mean was 2.9 days) in the Group-B. The mean of the post-operative pain (visual analogue scale) after six hours of the operation was similar in both groups i.e., 4.6 and 4.5 in Group-A and Group-B respectively, but Group-A patients experienced more pain (mean pain score six) and movement limitations as compared to Group-B (mean pain score 4.5) during overall hospital course. During the follow-up period, there were no differences in pain and movements. The patients in both the groups were able to return to their routine activities by the 2^nd^ week of the operation.

Three (11.5%) patients developed postoperative seroma between the prosthetic mesh and the abdominal wall and one (3.8%) small hematoma in Group-A, whereas two (7.1%) postoperative seroma and no hematoma in Group-B. All these patients were managed conservatively (Table-II).

**Table II:** Operative data.

Variable	Group-A	Group-B	p-value
Mean Anesthetic time (minutes)	98.6	77.3	< 0.01
Mean Operating time (minutes)	75.4	66.5	< 0.01
Mean Post-operative pain score at 6 hours	4.6	4.5	> 0.01
Mean Post-operative pain at 24 hours (VAS)	6	4.5	> 0.01
Mean Hospital stay (range) days	3.3 (2 – 5)	2.9 (2 – 4)	> 0.01
Mean Return to daily activities (days)	8	9	> 0.01
Mean Return to work (day)	21	19	> 0.01
Post-operative Complications:			
• Seroma	3	2	> 0.01
• Hematoma	1	0	> 0.01
• Prolonged ileus	1	0	> 0.01

Follow-up involved a physical assessment, ultrasound examination if needed at the outpatient clinic after one week, followed by monthly assessment for the first six months, then every three months up to the end of the study. The mean length of follow-up was 16±8 months (range: 12 – 30 months). No recurrence of the umbilical hernia was observed in all the patients.

## DISCUSSION

Generalized obesity and abdominal obesity i.e., intra-abdominal fat accumulation is considered as major health problem worldwide.^8^ Waist circumference is now a standard for the diagnosis of metabolic syndrome (abdominal fat and its metabolic consequences) and the average waist circumference is increasing globally.^9^,^12^

The complications observed in open repair of umbilical hernia with mesh, enforces to adopt laparoscopic technique and this approach is gaining popularity all over the world. Since the first report of laparoscopic ventral hernia repair in 1993 by Le Blanc K et al^10^, this is the first prospective study to our knowledge that explores the outcome of laparoscopic umbilical hernia repair in patients with abdominal obesity

Previous studies showed the negative impact of obesity on surgical outcomes generally^6^,^13^,^14^ and they described obesity as a risk factor for the development of umbilical hernias as well as for recurrence and complications after hernia repair.^15^ However, to our knowledge, this is the one among very few studies to examine the effect of abdominal obesity as defined by abnormal waist circumference on the outcome of patients undergoing laparoscopic umbilical hernia repair. Many published series showed that the obese patients in general are more likely to have longer surgery duration as compared to the patients with normal weight for a number of reasons.^16^ In this study, it is observed that the mean BMI in the abdominal obesity patients (Group-A) is more than Group-B (Table-I) and they had a longer duration of operation as compared to Group-B. We think that the increased duration of operative time is due to the following reasons. Introducing the trocars is difficult due to the excess adiposity and to overcome this problem, we used the bladeless optical trocars. Secondly, the excess of intra-abdominal fat in obese patients make mobilization of the bowel and handling the mesh intra-peritoneally a complicated task. To resolve this issue, more time was spent to prevent any complication. This is consistent with the results of many similar studies and the overall rate of complications was similar in both groups in these studies.^17^ Moreover, the incidence of seroma formation was low as compared to other studies^18^ and no recurrence was observed.

The mean duration of post-operative ileus was 16.3 hours in Group-A patients, while it was 15.9 hours in Group-B. One (3.8%) patient in the Group-A suffered from prolonged ileus (36 hours), and this is consistent with the results of many reports claiming prolonged ileus in 1 to 3% of laparoscopic ventral hernia repairs.^19^-^21^ Group-A patients experienced more pain (mean pain score 6) and movement limitations as compared to Group-B (mean pain score 4.5) during hospital course, but there were no differences during the follow-up period. However, a prospective study conducted in Pakistan showed better overall results of laparoscopic para-umbilical hernia repair as compared to the conventional open technique.^22^

### Limitation

The limitation of the study is that it was done in male patients only.

## CONCLUSION

Though the laparoscopic umbilical hernia repair is more challenging in patients with an abnormal waist circumference, yet it is feasible. Patients with an abdominal obesity have longer anesthetic and operative time but have a similar complication profile as compared to the patients without abdominal obesity.

### Authors’ contribution:

**Al-Mulhim AS:** Analyzed, edited, reviewed and finally approved the manuscript.

**Memon AQ:** Searched and collected the data, analyzed and wrote the manuscript, he is also responsible for the accuracy of study.
